# Rare metastasis of nasopharyngeal carcinoma to the thyroid gland with subsequent metastatic abdominal lymph nodes: A case report and literature review

**DOI:** 10.1097/MD.0000000000008373

**Published:** 2017-11-10

**Authors:** Changjing Cai, Hong Shen, Wenqiang Liu, Junli Ma, Yan Zhang, Ling Yin, Jindong Li, Liangfang Shen, Shan Zeng

**Affiliations:** a Department of Oncology; b Institute of Medical Sciences, Key Laboratory for Molecular Radiation Oncology of Hunan Province, Xiangya Hospital, Central South University, Changsha, Hunan, China.

**Keywords:** abdominal lymph nodes, nasopharyngeal carcinoma(npc), radio-chemotherapy, rarely metastasis, thyroid gland

## Abstract

**Rationale:**

Thyroid metastasis from nasopharyngeal carcinoma is rare. Metastasis of nasopharyngeal carcinoma to the thyroid gland with subsequent metastatic abdominal lymph nodes hasn’t been reported before. We want to share our experience about the treatment choice.

**Patient concerns:**

A 27-year-old man was diagnosed with nasopharyngeal nonkeratinizing carcinoma in August 2004. In March 2013 he underwent a thyroid carcinoma radical operation, and histological examination revealed metastasis to the thyroid gland from nasopharyngeal carcinoma. An 18F-FDG-PET/CT scan and biopsy showed metastatic abdominal lymph nodes of nasopharyngeal carcinoma in April 2015.

**Diagnoses:**

A 27-year-old man was diagnosed with metastasis of nasopharyngeal carcinoma to the thyroid gland with subsequent metastatic abdominal lymph nodes.

**Interventions:**

The patient was treated with concurrent chemotherapy and radiotherapy for nasopharyngeal carcinoma and metastasis to the thyroid gland. The metastases to the abdominal lymph nodes received chemotherapy.

**Outcomes:**

After 6 cycles of chemotherapy with gemcitabine, cisplatin, and 5-fluorouracil for metastasis to the abdominal lymph nodes, the patient is currently asymptomatic with stable disease and improved quality of life.

**Lessons:**

The treatment choice for metastasis of nasopharyngeal carcinoma depends on the clinical disease extent, and surgery and/or chemo-radiation therapy must be drafted to the individual patient in order to improve the prognosis and quality of life.

## 1 Introduction

Metastasis of nasopharyngeal carcinoma in thyroid gland with subsequent metastatic abdominal lymph nodes has not been reported before. In August 2004, a 27-year-old man was diagnosed with nasopharyngeal low differentiated squamous carcinoma; in March 2013, he underwent a thyroidectomy and histological examination revealed metastasis in thyroid gland; in April 2014 an 18F-FDG-PET/CT scan and biopsy showed metastatic abdominal lymph nodes of nasopharyngeal carcinoma. Treated with concurrent radiochemotherapy for nasopharyngeal carcinoma and metastasis in thyroid gland, and chemotherapy for the subsequent metastatic abdominal lymph nodes, the patient is asymptomatic with stable disease and improved quality of life.

## 2 Case presentation

**Figure 1:**
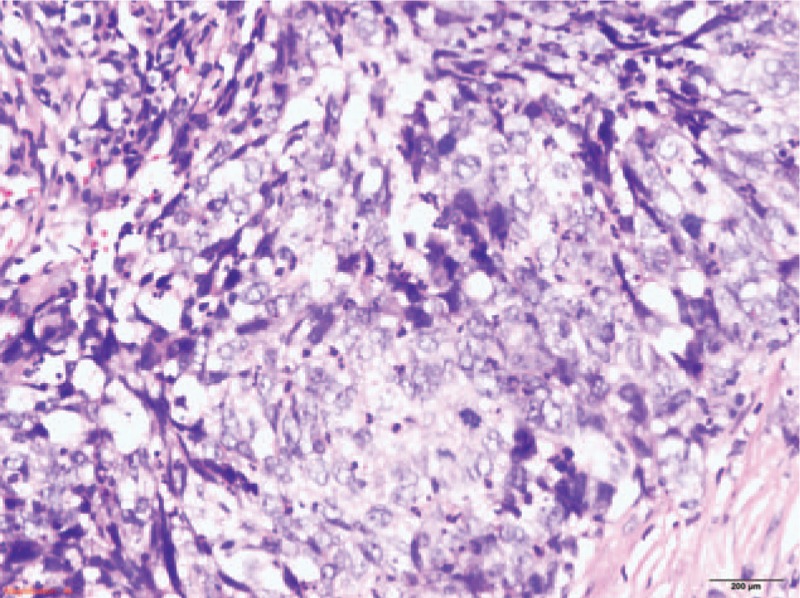
Pathologic diagnosis: primary nasopharyngeal nonkeratinizing carcinoma.

A 27-year-old Chinese male was hospitalized after he found asymptomatic lumps on the right side of his neck in July 2004. Enhanced computed tomography (CT) scan showed an abnormality in his nasopharynx. The histological examination of the biopsy revealed a nonkeratinizing carcinoma (Fig. 1). Clinical staging according to the UICC classification of 2002 was cT3, N2, M0. The patient underwent concurrent radiochemotherapy with oral tegafur 300 mg 3 times per day, 7 days a week, from August 9, 2004 to September 24, 2004. Radiotherapy was delivered with cobalt-60 for a total dose of 34 Gy in 17 fractions to the facial-cervical and supraclavicular fields, 50 Gy in 25 fractions to the auriculotemporal field bilaterally and the anteriorcervical field followed by 60Gy in 30 fractions to the left cervical region, and 70 Gy in 35 fractions to the right upper cervical field with 9 million electron volts (Mev)—β. After the concurrent treatment, the patient was in complete remission (CR) of the disease.

**Figure 2:**
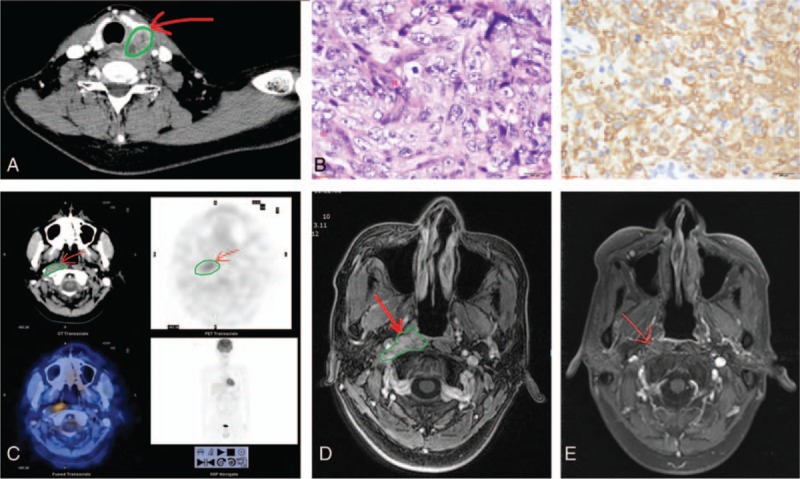
(A) A contrast-enhanced computed tomography scan observed heterogeneous enhancement of the 28 mm × 18 mm mass in the left lobe of the thyroid gland. (B) A thyroid metastasis from nasopharyngeal carcinoma: a low differentiated squamous carcinoma in histological examination (left) and cytokeratin 5/6 (++) in immunohistochemistry staining (right). (C) Recurrent nasopharyngeal carcinoma shown in 18F-flurodeoxyglucose positron emission computerized tomography. (D) Recurrent nasopharyngeal carcinoma shown in magnetic resonance imaging. (E) Remission of recurrent nasopharyngeal carcinoma after concurrent chemoradiotherapy.

Follow-up visits were negative until April 2013 when, 105 months from the initial diagnosis, the patient found an asymptomatically hard mass on the left side of his neck. There were no symptoms of hypo- or hyperthyroidism. Upon arrival at the hospital, cervical ultrasound showed a 27 mm × 25 mm hypoechoic mass with obscure boundaries and irregular shape in the left lobe of the thyroid gland. Sand-like hyperechoic-foci were visible in the hypoechoic mass. Color doppler flow imaging showed strip blood flow in the mass. Numerous hypoechoic nodes (the largest one was 11 mm × 10 mm) were detected in the left side of his neck. A tracheal deviation was observed without trachyphonia. An unenhanced CT scan showed an ill-defined, low-density mass measuring 28 mm × 18 mm in the left lobe of the thyroid gland. On contrast-enhanced CT scan, heterogeneous enhancement in the mass was observed, with 41 Hounsfield units (HU) before contrast enhancement, and 92 HU after contrast enhancement images, respectively (Fig. 2A). Preoperative serum thyroid-stimulating hormone (TSH) was elevated (6.920 mIU/L, reference range: 0.27–4.2 mIU/l). Free triiodothyronine was 3.630 pmol/L (2.8–7.1 pmol/L), free thyroxin was 14.900 pmol/L (12–22 pmol/L), anti-thyroglobulin antibodies were 28.360 IU/mL (0–115.00 IU/mL), and anti-thyroid peroxidase antibodies were 25.020 IU/mL (0–34.00 IU/mL). A total thyroidectomy of the left lobe, a subtotal thyroidectomy of the right lobe, and a left recurrent laryngeal nerve exploratory operation were performed on April 23, 2013. There was an extremely hard mass closely adhering to the surrounding tissues in the left lobe of the thyroid gland. Part of the right lobe of the thyroid gland and the upper portion of the left recurrent laryngeal nerve were invaded. Following a diagnosis of low differentiated carcinoma by a frozen section examination during the operation, the pathologist finally reported low differentiated squamous carcinoma. Immunohistochemistry showed cytokeratin 5/6 (+ +), cytokeratin high (++), cytokeratin low (−), tumor protein 63 (++), thyroid transcription factor-1 (−), thyroglobulin (−), synaptophysin (−), Chromogranin A (−), and Epstein–Barr encoded RNAs, (EBER) (+) (in situ hybridization) (Fig. 2B). Histological examination excluded a primary tumor of the thyroid gland, but revealed a secondary lesion from nasopharyngeal carcinoma (NPC). In the meantime, an 18F-flurodeoxyglucose positron emission computerized tomography (18F-FDG PET/CT) scan showed recurrent NPC without other distant lesions that resulted in an increased FDG uptake with SUV_max_ 3.9 (Fig. 2C). The same results were achieved by magnetic resonance imaging (MRI) (Fig. 2D). After cardiological and renal evaluation, the patient received 3-dimensional conformal and intensity-modulated radiation therapy from May 30 to July 17, 2013. The doses to the planning target volumes of the primary tumor, the involved lymph nodes and high risk region, and the uninvolved regional nodal areas were 67.2 Gy, 70.4 Gy, and 60.8 Gy, respectively, and were delivered simultaneously over 32 fractions. There were 4 cycles of chemotherapy with docetaxel 80 mg/m^2^on day 1 every 3 weeks and nedaplatin 85 mg/m^2^on day 2 every 3 weeks from June 1 to June 5, September 2013. At the end of the forth cycle of chemotherapy, MRI of the nasopharynx showed a partial remission (Fig. 2E).

**Figure 3:**
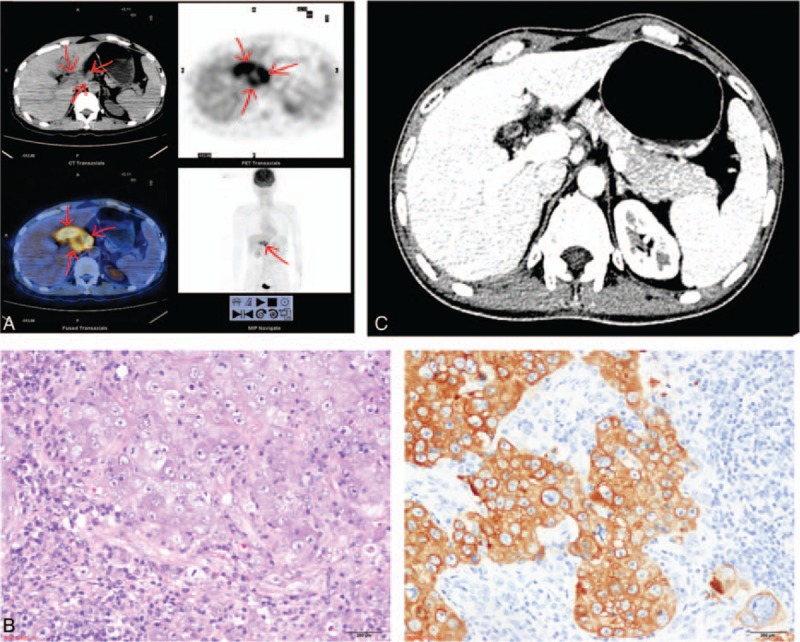
(A) Metastatic hepatic hilum and the retroperitoneal lymph nodes from nasopharyngeal carcinoma shown in 18F-flurodeoxyglucose positron emission computerized tomography. (B) The biopsy of the abdominal lymph nodes: low differentiated squamous carcinoma (left) and Pan-cytokeratin (+) in immunohistochemistry staining. (C) A contrast-enhanced CT scan of the abdominal lymph nodes after 6 cycles of chemotherapy.

On April 2015, this patient was hospitalized again because of abdominal pain. The enhanced CT showed several enlarged lymph nodes around his hepatic hilum, pancreas, and retroperitoneum. The minor axis of the largest lymph node was 2.1 cm. An 18F-FDG PET/CT scan did not show any other abnormal hypermetabolicareas except the hepatic hilum and the retroperitoneal lymph nodes, which showed an increased FDG uptake with SUV_max_ 7.4 (Fig. 3A). The biopsy of these lymph nodes showed low differentiated squamous carcinoma, and immunohistochemistry showed Pan-cytokeratin (+) (Fig. 3B) and EBER(+) (in situ hybridization). It was diagnosed as metastatic hepatic hilum and retroperitoneal lymph nodes from NPC. This patient received 6cycles of chemotherapy with gemcitabine1000 mg/m^2^on day 1 and day 8 every 3 weeks, cisplatin 25 mg/m^2^on days 1 to 3 every 3 weeks, and 5-fluorouracil 600 mg/m^2^/day continuous infusion for 120 hours every 3 weeks from March 31, to August 17, 2015. During chemotherapy, the patient presented clinical symptoms and signs including dental ulcer, odynophagia, nausea, vomiting, leucopenia, and weight loss. The abdominal pain disappeared after 1 cycle of the treatment. All enlarged lymph nodes disappeared except several tiny retroperitoneal lymph nodes after 6 cycles of chemotherapy (Fig. 3C). Currently, the patient is asymptomatic with stable disease and improved quality of life.

## 3 Discussion and literature review

Thyroid metastasis from NPC has been reported in only 4 cases in the literature, and its treatment is not systematized in the literature. Jalaludin et al reported the first case of metastatic thyroid carcinoma from NPC (54-years’ old) in 1994. The patient was diagnosed with NPC and received radiotherapy to the neck and post-nasal space. One month after completing radiotherapy, she presented with a neck mass. Fine needle aspiration cytology of the neck mass was interpreted as “poorly differentiated squamous cell carcinoma,” and an emergency tracheostomy was performed because of difficulty breathing with stridor at rest 1 week later. Histopathological examination of the tumor showed an undifferentiated carcinoma infiltrating normal-appearing thyroid follicles. The patient died while she was on home leave before receiving further radiotherapy to the thyroid gland ^[1]^.

The second case concerned a 50-year-old patient treated for an undifferentiated NPC with chemoradiotherapy. Four years after the primary treatment, he presented with a compressive thyroid mass with cervical lymph node involvement.^[2]^ The third case reported a 59-year-old male who was diagnosed with nasopharyngeal infiltrating spinocellular carcinoma. He received cisplatin and 3D conformal radiotherapy. Thirty months later, a CT scan showed progressive disease with a thyroid mass infiltrating the trachea. A subtotal thyroidectomy of the right lobe and a mediastinal tracheostomy were performed because of respiratory failure. Histological examination revealed metastatic thyroid carcinoma from NPC. After 6 cycles of chemotherapy (taxotere 75 mg/m^2^ every 3weeks and gemcitabine 800 mg/m^2^ on days 1–8 every 3 weeks), an 18F-FDG-PET/CT scan showed CR.^[3]^

Chen et al investigated 3957 patients with thyroid cancer between 1977 and 2012. There were 56 patients (1.4%) diagnosed with metastatic cancers to the thyroid (MCT). Of the MCT, metastasis of lung cancer (35.7%) to the thyroid was the most common category. Other primary sites of MCT were the head and neck (14.3%, including 1 case with metastasis of NPC to the thyroid gland), gastrointestinal tract (21.4%), kidneys (8.9%), breast (5.4%), cervix (3.6%), and unknown primary site (10.7%). Twelve patients were diagnosed with metachronous thyroid metastases after the primary cancer, with interval periods of 11 to 102 months between diagnosis of primary and metastatic cancer. The mean 5-, 10-, 20-, and 60-month survival rates were 46.4, 32.1, 21.4, and 7.9%, respectively, for these patients. Patients with metachronous thyroid carcinoma had significantly better survival than patients with synchronous cancer.^[4]^

In 1996, Huang et al. reported that the frequencies of distant metastases in nasopharyngeal carcinoma were as follows: bone (75% of total metastatic patients), lung (46%), liver (38%), and retroperitoneal lymph nodes (10%). Most of the distant metastases (95%) occurred within 3 years after completion of radiotherapy: the first year (52%), the second year (23%), and the third year (20%).^[5]^

To our knowledge, there has been no reported case of a metastasis of nasopharyngeal carcinoma to the thyroid gland with subsequent metastatic abdominal lymph nodes. In our case, the patient was diagnosed with metachronous thyroid metastases after the primary cancer, with an interval of 105 months between diagnosis of primary and metastatic cancer. Metastatic abdominal lymph nodes were found 11 months after metastasis to the thyroid gland. The patient is now asymptomatic with an improvement in quality of life and stable disease after treatment.^[4]^

According to the literature, a patient with NPC should receive follow-up care for up to 9 years after the primary diagnose. 18F-FDG-PET/CT is a feasible way to detect metastases from nasopharyngeal carcinoma. Therapeutic decision-making depends on the clinical disease extent, and surgery and/or chemoradiation therapy must be drafted to the individual patient to improve the prognosis and quality of life. For patients with subsequent metastatic abdominal lymph nodes after metastasis of nasopharyngeal carcinoma to the thyroid gland, we recommend chemotherapy with gemcitabine 1000 mg/m^2^on day 1 and day 8 every 3 weeks and cisplatin 25 mg/m^2^on days 1 to 3 every 3 weeks and 5-fluorouracil 600 mg/m^2^/day continuous infusion over 120 hours every 3 weeks. The change of chemotherapy regimen and/or additional target therapy should be considered while a poor curative effect of chemotherapy was achieved.
